# Comparative Effects of Spinal Anesthesia and Combined Spinal with Peripheral Nerve Blocks on Postoperative Outcomes in Anterior Cruciate Ligament Repair

**DOI:** 10.3390/jcm13226845

**Published:** 2024-11-14

**Authors:** Sanja Berić, Tamara Murselović, Mark Žižak, Stjepan Bulat, Goran Vrgoč

**Affiliations:** 1 Clinical Hospital Sveti Duh, Sveti Duh 64, 10000 Zagreb, Croatia; sanja.beric1@gmail.com (S.B.); m_zizak@hotmail.com (M.Ž.); bulatstjepan@gmail.com (S.B.); goran.vrgoc@kif.unizg.hr (G.V.); 2 Faculty of Dental Medicine and Health, Josip Juraj Strossmayer University of Osijek, Crkvena 21, 31000 Osijek, Croatia; 3 Faculty of Kinesiology, University of Zagreb, Horvaćanski zavoj 15, 10110 Zagreb, Croatia

**Keywords:** arthroscopic ACL reconstruction, spinal anesthesia, adductor canal block, sciatic nerve block, postoperative pain management, orthopedic surgery

## Abstract

**Objectives**: This study aimed to compare the effectiveness of spinal anesthesia (SA) alone versus combined spinal anesthesia with adductor canal block (ACB) and sciatic nerve block (SNB) (SA + ACB + SNB) in patients undergoing arthroscopic anterior cruciate ligament (ACL) reconstruction. We hypothesized that SA + ACB + SNB would provide better analgesia, greater patient satisfaction, and shorter postanesthesia recovery times than SA alone. **Methods**: A prospective randomized controlled trial was conducted with 60 patients aged 15–49 years scheduled for elective arthroscopic ACL reconstruction. Participants were randomly assigned to receive either SA or SA + ACB + SNB. Postoperative pain was assessed using the Visual Analog Scale (VAS) at 4, 12, and 24 h post-operation. General health was evaluated using the 12-item Short Form Survey (SF-12) at 1 month postoperatively. Range of motion and analgesic consumption were also recorded. **Results**: The median VAS score at 4 h post-operation was significantly lower in the SA + ACB + SNB group compared to the SA group (0 [IQR: 0–1] vs. 2 [IQR: 1–3], *p* = 0.0137). No significant differences in VAS scores were found at 12 h (*p* = 0.9282) and 24 h (*p* = 0.5809). PCS-12 and MCS-12 scores did not differ significantly between groups. The SA group had a lower postoperative range of motion (ROM) compared to the SA + ACB + SNB group, with a mean active ROM of 40.67 degrees (±23.52) versus 72.17 degrees (±24.69), respectively (*p* < 0.0001). Analgesic consumption was similar, with 53.33% of participants in each group using postoperative analgesics (*p* = 1.0). The mean surgery duration was 74.6 min. The gender distribution was 83% male and 17% female, with an average age of 27.7 years. **Conclusions**: Adding ACB and SNB to spinal anesthesia improved immediate postoperative pain relief and preserved range of motion in patients undergoing ACL reconstruction, suggesting potential clinical benefits in pain management and functional recovery.

## 1. Introduction

Anterior cruciate ligament (ACL) reconstruction is a common orthopedic procedure aimed at restoring knee stability and function following ACL rupture. However, despite advances in surgical techniques, postoperative pain management remains a critical challenge that significantly impacts patient recovery and rehabilitation outcomes. Effective pain control not only enhances patient comfort but also facilitates earlier mobilization and reduces the risk of long-term complications such as joint stiffness and chronic pain [1,2].

Traditionally, various anesthesia techniques have been employed to manage pain after ACL reconstruction. Spinal anesthesia and femoral nerve blocks are among the most frequently used methods, each offering specific benefits and limitations. Spinal anesthesia provides excellent pain relief but lacks targeted action at the site of surgery, which femoral nerve blocks can achieve by directly blocking pain transmission from the knee [3]. Studies have shown diverging results on the efficacy of combining spinal anesthesia with peripheral nerve blocks, with some reporting enhanced pain relief and others indicating minimal additional benefits [4].

Spinal anesthesia is a regional anesthesia technique used to achieve anesthesia of the lower part of the body by injecting a local anesthetic into the subarachnoid space, where it mixes with cerebrospinal fluid. Its advantages include rapid onset of action, reliable and effective analgesia, lower risk of complications compared to general anesthesia, and reduced need for opioids in the postoperative period. Possible complications of this type of anesthesia include hypotension, bradycardia, and post-dural puncture headache [5].

The adductor canal block (ACB) is a regional anesthesia technique increasingly used in orthopedic surgeries, particularly for knee procedures. This block targets the saphenous nerve and other distal branches of the femoral nerve, providing pain relief while preserving quadriceps strength. This muscle strength preservation is a crucial advantage over the femoral nerve block (FNB), which often causes significant muscle weakness, delaying mobilization, and increasing the risk of falls after surgery [6]. Several studies have shown that ACB provides analgesia comparable to FNB but with less quadriceps weakness [7,8]. Additionally, the use of ultrasound increases the success rate of ACB by allowing precise visualization of the adductor canal, facilitating accurate delivery of the local anesthetic. This technique has become a cornerstone of multimodal analgesic regimens, promoting faster postoperative recovery and shortening hospital stays [5]. Blocking these nerves provides targeted knee analgesia, reducing the need for opioids and speeding up postoperative recovery [9].

The sciatic nerve is the largest and longest nerve in the body. It provides motor and sensory functions [10]. The sciatic nerve block (SNB) is a regional anesthesia technique used to achieve anesthesia of the lower extremities, particularly the leg, foot, and ankle. There are several approaches to performing a sciatic nerve block, including the posterior, lateral, and anterior approaches [6].

The knee joint has complex innervation, with anterior, medial, and posterior regions contributing to postoperative pain following ACL reconstruction. The combination of ACB and SNB allows for a complete sensory blockade of these regions, which can significantly reduce pain intensity and duration in the early postoperative phase compared to spinal anesthesia alone or with a single block. However, studies report mixed results regarding the added benefits of combining spinal anesthesia with peripheral nerve blocks, with some findings supporting improved pain relief and others suggesting minimal additional benefits [11].

The main aim of this work is to conduct a comprehensive comparison of spinal anesthesia alone versus its combination with adductor canal nerve block and sciatic nerve block, examining their effectiveness in controlling postoperative pain and facilitating early physical therapy and mobilization. Although spinal anesthesia and nerve blocks like ACB or SNB are individually well-documented in orthopedic pain management, there is limited research on the combined use of these techniques specifically for ACL reconstruction. Existing studies on this multimodal approach in knee surgeries offer mixed results, particularly regarding the added benefit of combining peripheral nerve blocks with spinal anesthesia for ACL procedures. This lack of consensus creates a gap in the literature on the optimal anesthesia strategy for effective postoperative recovery in ACL reconstruction. This study is significant as it contributes to the ongoing debate and development of best practices for anesthesia in ACL reconstruction, ultimately aiming to improve patient outcomes.

We performed a prospective randomized controlled study comparing preoperative spinal anesthesia (SA) to combined spinal anesthesia with adductor canal block (ACB) and sciatic nerve block (SNB) in patients undergoing arthroscopic ACL reconstruction and semitendinosus and gracilis tendon harvest for ACL graft. We hypothesized that patients who received SA + ACB + SNB would have improved analgesia measured by lower pain scores on standardized pain scales (e.g., VAS) post-treatment compared to baseline or control, longer-lasting pain relief, and reduced need for additional analgesics, as well as greater patient satisfaction.

## 2. Materials and Methods

This prospective randomized controlled study was conducted to compare preoperative spinal anesthesia (SA) to combined spinal anesthesia with adductor canal block (ACB) and sciatic nerve block (SNB) in patients undergoing arthroscopic ACL reconstruction. Only patients with isolated ACL injuries without any chondral damage or meniscal injuries were included in the study. All patients demonstrated positive findings on magnetic resonance imaging (MRI), as well as positive results on the anterior drawer test and Lachman test, confirming anterior cruciate ligament (ACL) rupture.

Ethical approval for the study was obtained from Institutional Review Board (IRB) approval (Approval No. 03-6544), and all participants provided written informed consent. Participants included in the study were adults aged 15–49 years undergoing elective arthroscopic ACL reconstruction with m. semitendinosus and m. gracilis tendon graft. By including patients as young as 15, we aim to capture a representative sample of the population most commonly affected by ACL injuries. Younger patients, especially those involved in sports, have specific recovery and rehabilitation needs, such as maintaining muscle strength, range of motion, and quick return to physical activity. Examining postoperative pain management techniques in this age group is therefore essential for identifying effective approaches that meet their unique demands for fast, functional recovery. Exclusion criteria were a history of chronic pain, previous knee surgery, open ACL reconstruction, contraindications to regional anesthesia, or inability to understand the study protocol.

Patients in this study were randomly assigned by an independent study coordinator to one of two groups: spinal anesthesia alone (SA) or spinal anesthesia combined with adductor canal and sciatic nerve blocks (SA + ACB + SNB). Due to the nature of the interventions, patients were aware of whether they received spinal anesthesia alone or the additional peripheral nerve blocks (ACB and SNB). Therefore, complete blinding of patients was not feasible in this study. To minimize potential bias in data collection, the doctor responsible for recording primary and secondary outcomes—postoperative pain scores (VAS), range of motion (ROM), opioid consumption, and quality of life (SF-12)—was blinded to the patients’ group assignments. This approach aimed to ensure that all outcome measurements remained objective and unaffected by knowledge of the anesthesia technique used.

Patients in the SA group received spinal anesthesia with 0.5% levobupivacaine 15 mg.

Patients in the SA + ACB + SNB group received spinal anesthesia as described above, along with an adductor canal block and sciatic nerve block under ultrasound guidance. The adductor canal block was performed using 0.5% levobupivacaine 10 mL, and the sciatic nerve block was performed using 0.25% levobupivacaine 20 mL. Ultrasound was used for all peripheral nerve blocks. A total of four anesthesiologists were involved in the study. Only one anesthesiologist performed all peripheral nerve blocks (ACB and SNB) to maintain consistency in technique. The spinal anesthesia was administered by three different anesthesiologists, each trained in the procedure, ensuring standardized application across patients.

All patients underwent arthroscopic ACL reconstruction performed by a single surgeon using a standardized technique. For ACL reconstruction, the m. semitendinosus and m. gracilis tendons were harvested from the pes anserinus through a 2 cm vertical incision. The tendons were subsequently prepared on a graft preparation station. A femoral tunnel was drilled through the anteromedial portal, and a tibial tunnel was created using a tibial guide (Arthrex, Naples, FL, USA) set at an angle of 55 degrees. Proximal fixation of the ACL graft was achieved with the ACL TightRope II device (Arthrex, Naples, FL, USA), while distal fixation was secured at 20 degrees with a FastThread Biocomposite Interference Screw (Arthrex, Naples, FL, USA). Following placement and fixation of the graft, additional tightening was performed with the ACL TightRope II. At the end of the procedure, graft impingement and graft tension were assessed using a probe, and the positions of the screw and endobutton were verified via intraoperative X-ray imaging in the operating room. The duration of surgery was recorded from skin incision to skin closure. Esmarch was used in all patients to minimize bleeding. After surgery, bulky bandages were applied and left in place until the following morning, at which time they were removed and replaced with lighter bandages to facilitate range of motion (ROM) measurement. ROM was assessed before surgery and 20–24 h postoperatively, ensuring that measurements were taken without bulky bandages.

The primary outcome measure was postoperative pain, assessed using the Visual Analog Scale (VAS) at 4 h, 12 h, and 24 h post-surgery. Secondary outcomes included patient-reported general health assessed using the 12-item Short Form Survey (SF-12) version 1.0 at 1-month post-surgery, range of motion, and postoperative opioid consumption.

VAS scores were recorded at specified time points post-surgery. SF-12 scores were collected at 1 month postoperatively. The range of motion was measured using a goniometer preoperatively and postoperatively. Analgesic consumption was recorded based on patient medical records.

Data were analyzed using IBM SPSS Statistics version 29.0.2.0. The normality of continuous variables was assessed using the Shapiro–Wilk test. Independent t-tests were used for normally distributed variables, while Mann–Whitney *U* tests were applied for non-normally distributed variables. Chi-square tests were used to compare categorical variables. Statistical significance was set at *p* < 0.05.

## 3. Results

### 3.1. Participant Characteristics

A total of 60 patients were included in the study, divided into two groups based on the type of anesthesia administered: spinal anesthesia (SA) and combined spinal anesthesia with adductor canal block and sciatic nerve block (SA + ACB + SNB). The gender distribution of the study participants was 83% male and 17% female, with an average age of 27.7 years (15–49).

### 3.2. Visual Analog Scale (VAS) Pain Scores

**Figure 1:**
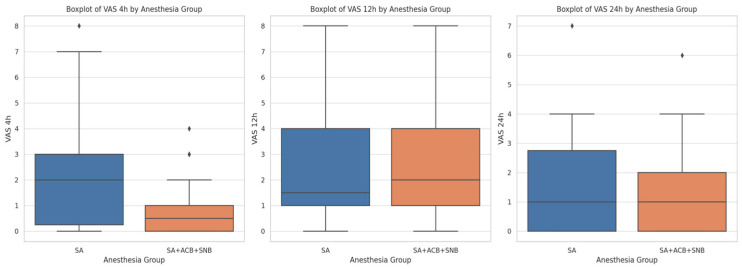
The boxplot displays the distribution of Visual Analog Scale (VAS) pain scores at 4 h, 12 h, and 24 h post-operation for two anesthesia groups: spinal anesthesia (SA) and combined spinal anesthesia with adductor canal block and sciatic nerve block (SA + ACB + SNB).

The VAS pain scores were assessed at 4 h, 12 h, and 24 h post-operation. The median VAS scores for the SA group were higher compared to the SA + ACB + SNB group at 4 h post-operation. Specifically, the median VAS scores at 4 h were 2 (IQR: 1–3) for the SA group and 0 (IQR: 0–1) for the SA + ACB + SNB group, showing significantly lower pain levels in the SA + ACB + SNB group (*p* = 0.0137). However, there were no significant differences in VAS scores between the two groups at 12 h (*p* = 0.9282) and 24 h (*p* = 0.5809) post-operation. The boxplots illustrate that the combined anesthesia technique (SA + ACB + SNB) provides better pain control in the immediate postoperative period (4 h), but pain levels between the groups become similar by 12 and 24 h post-operation (Figure 1).

### 3.3. SF-12 Health Survey Scores

**Figure 2:**
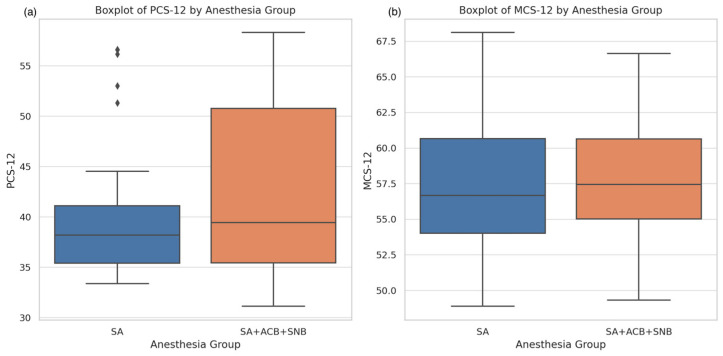
Boxplots for PCS-12 and MCS-12 scores by anesthesia group. (**a**) The left plot shows the distribution of PCS-12 scores for each group (SA and SA + ACB + SNB); (**b**) The right plot shows the distribution of MCS-12 scores for each group.

The physical component scores (PCS-12) and mental component scores (MCS-12) were evaluated using the SF-12 Health Survey. The PCS-12 scores for the SA group were lower than those for the SA + ACB + SNB group, as depicted in the boxplots (Figure 2). Despite this visual difference, the Mann–Whitney *U* test indicated no significant difference in PCS-12 scores between the two groups (*p* = 0.569). Similarly, the MCS-12 scores did not show a significant difference between the groups according to the independent *t*-test (*p* = 0.787).

### 3.4. Range of Motion

The active range of motion (ROM) for patients before and after the procedure was analyzed and compared between the two anesthesia groups: spinal anesthesia (SA) and spinal anesthesia combined with adductor canal block and sciatic nerve block (SA + ACB + SNB) (Table 1).

**Figure 3:**
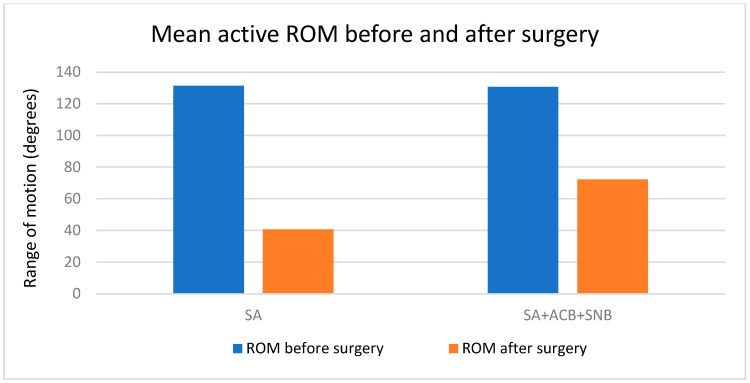
Mean active range of motion before and after surgery between the two groups visually demonstrates that while both groups experienced a decrease in ROM following surgery, the SA + ACB + SNB group retained a greater range of motion compared to the SA group.

The mean active ROM before the procedure was similar between the two groups. The SA group had a mean ROM of 131.33 degrees (±3.46), while the SA + ACB + SNB group had a mean ROM of 130.67 degrees (±2.54). The mean active ROM after the procedure was significantly different between the two groups. The SA group had a mean active ROM of 40.67 degrees (±23.52), whereas the SA + ACB + SNB group had a higher mean active ROM of 72.17 degrees (±24.69). The change in active ROM (after minus before) also showed significant differences. The SA group experienced a larger decrease in active ROM with a mean change of −90.67 degrees (±23.07). In contrast, the SA + ACB + SNB group had a mean change of −58.50 degrees (±25.43). A Mann–Whitney *U* test was conducted to compare the change in active ROM between the two groups. The test revealed a U-statistic of 189.5 and a *p*-value of 0.000094, indicating a statistically significant difference in the change in ROM between the SA and SA + ACB + SNB groups (*p* < 0.05) (Figure 3). The analysis indicates that patients in the SA + ACB + SNB group showed improved postoperative ROM compared to the SA group.

### 3.5. Analgesic Consumption

Postoperative analgesic consumption was systematically recorded based on patient-reported use of analgesics following surgery, guided by a standardized pain management protocol. Analgesic administration was structured according to Visual Analog Scale (VAS) pain scores to ensure consistent and objective pain management across both groups:

- VAS 0–3: Patients reporting pain in this range received no analgesic intervention.
- VAS 4–6: Patients with moderate pain (VAS 4–6) were administered a combination of intravenous nonsteroidal anti-inflammatory drugs (NSAIDs) and paracetamol.
- VAS > 7: For patients experiencing severe pain (VAS > 7), intravenous tramadol at a dose of 50–100 mg was provided.

Each patient’s postoperative pain scores and corresponding analgesic use were recorded by nursing staff and subsequently confirmed through patient reports during follow-up assessments. Both groups had an equal proportion of patients (53.33%) using postoperative analgesics. The chi-square test confirms that this difference is not statistically significant (*p* = 1.0), indicating that the type of anesthesia did not affect the likelihood of patients using analgesics postoperatively.

### 3.6. Surgery Duration

The mean surgery duration for all patients was 74.6 min, with no significant differences noted between the groups.

## 4. Discussion

This study aimed to compare spinal anesthesia (SA) alone versus combined spinal anesthesia with adductor canal block (ACB) and sciatic nerve block (SNB) (SA + ACB + SNB) in patients undergoing arthroscopic anterior cruciate ligament (ACL) reconstruction with m.semitendinosus and m.gracilis tendons harvesting for ACL graft. The results showed that the SA + ACB + SNB group experienced significantly lower immediate postoperative pain (4 h post-operation) compared to the SA group. Additionally, the SA + ACB + SNB group exhibited better preservation of active range of motion after knee ACL reconstruction postoperatively. There were no significant differences in pain scores at 12 and 24 h, SF-12 health survey scores, postoperative analgesic consumption, or surgery duration between the two groups.

Our findings are consistent with several previous studies demonstrating the benefits of regional anesthesia techniques in orthopedic surgeries. Naser et al. [12] reported that peripheral nerve blocks, such as the adductor canal block, provided better pain control than other methods in knee surgeries. Similarly, Sahin et al. [13] found that femoral nerve blocks significantly reduced postoperative morphine consumption and enhanced patient satisfaction following knee arthroplasty. Fowler et al. [11] also highlighted the superior analgesic efficacy of peripheral nerve blocks over epidural analgesia in major knee surgeries.

In the context of ACL reconstruction, Abdallah et al. [7] demonstrated that adductor canal block (ACB) provided noninferior analgesia and superior quadriceps strength compared to femoral nerve block (FNB). This aligns with our findings that SA + ACB + SNB improved pain control, especially in the first 4 h post-operation, without compromising functional outcomes. El Ahl [14] and Kamath and Faiaz [15] further supported the use of ACB in ACL reconstruction, noting its benefits in preserving quadriceps strength and facilitating early mobilization.

Our findings align with earlier research investigating the impact of different nerve blocks used alongside spinal anesthesia. For instance, a prospective double-blinded randomized study by Astur et al. [3] evaluated clinical outcomes using femoral nerve block with spinal anesthesia versus spinal analgesia alone in patients undergoing ACL reconstruction. They found that spinal anesthesia with a femoral nerve block provided significant pain relief shortly after surgery, but there were no additional benefits in pain control after the third postoperative day. This aligns with our findings where the SA + ACB + SNB group had superior pain relief at 4 h but showed no significant advantage at 12 and 24 h post-operation. Similarly, a randomized clinical trial by Harbell et al. [2] compared preoperative femoral nerve block alone versus combined femoral and sciatic nerve block for arthroscopic ACL reconstruction under general anesthesia. The study demonstrated improved analgesia, decreased opioid consumption perioperatively, and shorter PACU length of stay in the combined block group on the day of surgery. However, there was no significant difference in opioid consumption, pain scores, or patient satisfaction on postoperative days 1–3 between the groups. This suggests that the benefits of additional nerve blocks may be limited to the immediate postoperative period, which is in agreement with our observation that the SA + ACB + SNB group experienced better pain control at 4 h but not at later time points. The study by Frost et al. [1] evaluated the efficacy of intraoperative femoral nerve block in reducing postoperative pain following ACL hamstring reconstruction under general anesthesia. They found that femoral nerve block may reduce pain on the night of surgery, but the reduction might not be clinically significant. This further supports the notion that while nerve blocks can be effective in the immediate postoperative period, their benefits may diminish over time, as observed in our study.

The significant reduction in immediate postoperative pain observed in the SA + ACB + SNB group has important clinical implications. Effective early pain management can enhance patient comfort, reduce opioid consumption, and facilitate early mobilization and rehabilitation, ultimately improving overall recovery and patient satisfaction. The better preservation of the range of motion in the SA + ACB + SNB group is particularly crucial for functional recovery in ACL reconstruction.

For instance, Goyal et al. [16] showed that a combined femoral–obturator–sciatic nerve block provides superior postoperative pain scores and earlier mobilization compared to spinal anesthesia in patients undergoing arthroscopic ACL reconstruction. This supports our observation of better pain control and ROM in the immediate postoperative period with the combined block technique. It may be worth mentioning that a significant advantage of the ACB over the femoral block is the early activation of the quadriceps muscle, which is extremely important for the early initiation of physical therapy in patients after ACL reconstruction. This is especially important for professional athletes. The femoral block increases the risk of ACL re-injury within the first year following reconstruction [17]. Similarly, Montes et al. [18] compared sciatic–femoral nerve block with low-dose spinal anesthesia for outpatient arthroscopic knee surgery. Although knee arthroscopy is a significantly less complex surgery and causes less pain compared to arthroscopic anterior cruciate ligament reconstruction, the study found that combined nerve block offers satisfactory anesthesia with a clinical profile similar to that of low-dose spinal anesthesia and is associated with significantly lower pain scores during the first 6 postoperative hours, which parallels our findings of improved pain management at 4 h post-operation when using peripheral nerve blocks.

A systematic review by Secrist et al. [19] further corroborates these results by demonstrating that peripheral nerve blocks, when used in combination with spinal anesthesia, can reduce opioid requirements and enhance functional recovery. These studies collectively highlight the benefits of multimodal analgesia, particularly in orthopedic surgeries such as arthroscopic ACL reconstruction.

However, consistent with the findings of Astur et al. and Harbell et al. [2,3], our study did not find significant differences in pain scores at 12 and 24 h post-operation or in overall analgesic consumption. This suggests that while combined nerve blocks provide significant immediate postoperative benefits, their long-term efficacy might be limited. The variations in study designs, anesthesia protocols, and patient populations, as well as the relatively short follow-up period in our study, may contribute to these discrepancies.

A major strength of this study is the randomized controlled design, which minimizes selection bias and enhances the validity of the findings. The use of validated outcome measures (VAS and SF-12) adds robustness to the results. However, this study has limitations, including a relatively small sample size that may limit generalizability and a short follow-up period that precludes assessment of long-term outcomes.

Future research should focus on comparing different combinations of regional anesthesia techniques to optimize postoperative pain management and functional recovery in ACL reconstruction. Studies with longer follow-up periods are essential to evaluate the long-term benefits and potential complications of these techniques. Additionally, investigating the effects of these anesthesia methods in diverse patient populations, such as those with chronic pain or opioid tolerance, would provide valuable insights for personalized pain management strategies.

Overall, the combined use of spinal anesthesia with peripheral nerve blocks, specifically ACB and SNB, offers significant advantages in immediate postoperative pain control and range of motion, aligning with the broader evidence in the literature.

## 5. Conclusions

In conclusion, the addition of adductor canal block and sciatic nerve block to spinal anesthesia significantly improves immediate postoperative pain relief and preserves active range of motion in patients undergoing arthroscopic ACL reconstruction. Furthermore, using this combination of peripheral nerve blocks, we avoid motor inactivation of the quadriceps muscle, as seen with the use of a femoral nerve block, resulting in earlier quadriceps activation and faster recovery, which is of utmost importance in professional athletes. Further research is needed to confirm these results and investigate long-term outcomes.

## Figures and Tables

**Table 1:** Range of motion before and after surgery for different anesthesia types.

Group	ROM Before Surgery (Mean ± std *)	ROM Before Surgery (Median)	ROM After Surgery (Mean ± std)	ROM After Surgery (Median)	Change (Mean ± std)	Change (Median)
SA	131.33 ± 3.46	130.0	40.67 ± 23.52	30.0	−90.67 ± 23.07	−100.0
SA + ACB + SNB	130.67 ± 2.54	130.0	72.17 ± 24.69	80.0	−58.50 ± 25.43	−50.0

* Standard deviation.

## Data Availability

The original contributions presented in the study are included in the article; further inquiries can be directed to the corresponding author.
